# Poultry Relief? Organic Farming May Reduce Drug Resistance

**DOI:** 10.1289/ehp.119-a489b

**Published:** 2011-11-01

**Authors:** Wendee Holtcamp

**Affiliations:** Houston-based writer Wendee Holtcamp has written about science and conservation for 15 years, publishing in *Miller-McCune*, *Nature*, *National Wildlife*, and other magazines.

Organic poultry is one of the fastest growing segments of the U.S. organic foods market, and many conventional poultry farms have converted to organic methods to capitalize on this demand. Among other requirements, organic growers are not allowed to use any antibiotics on chickens from hatching to slaughter. A new study indicates organic practices may help reduce the prevalence of antibiotic-resistant bacteria [*EHP* 119(11):1622–1628; Sapkota et al.].

A growing awareness of antibiotic resistance is part of the reason for the increased consumer demand for organic meat. Conventional food animal producers rely on antibiotics not only for treating sick animals but also for nontherapeutic purposes, such as disease prevention and growth promotion. This routine use of antibiotics has been linked to the rise of antibiotic-resistant bacteria, which can be passed along to people from contaminated food and through occupational exposure. According to the World Health Organization, the rate at which bacterial strains are developing resistance to antibiotics far outpaces the rate at which scientists are developing new medicines that can kill the strains.

A team of researchers from the University of Maryland School of Public Health isolated *Enterococcus* bacteria from feed, water, and litter collected at five conventional and five organic poultry farms in the mid-Atlantic United States. All the organic farms had recently converted from conventional techniques and were raising their first flock of organic broilers.

Of the *Enterococcus* bacteria collected at the various farms, 46% were *E. faecalis* and 43% were *E. faecium*, with the remainder being three less common species. There were no differences in bacterial prevalence between farm types. After isolating and growing the two primary species on agar, the researchers tested them for susceptibility to 17 antimicrobial agents.

Compared with those collected from conventional farms, *E. faecalis* strains collected from organic poultry houses were less likely to be resistant to 9 antimicrobials, with significant differences for the compounds erythromycin and tylosin. *E. faecium* strains from organic farms were less likely to be resistant to 11 antimicrobials, with significant differences for 5 compounds: ciprofloxacin, gentamicin, nitrofurantoin, penicillin, and tetracycline. The researchers also documented that the percentages of multidrug-resistant bacteria were significantly lower among bacterial isolates recovered from organic versus conventional farms (10% vs. 42% for *E. faecalis*, and 17% vs. 84% for *E. faecium*).

**Figure:**
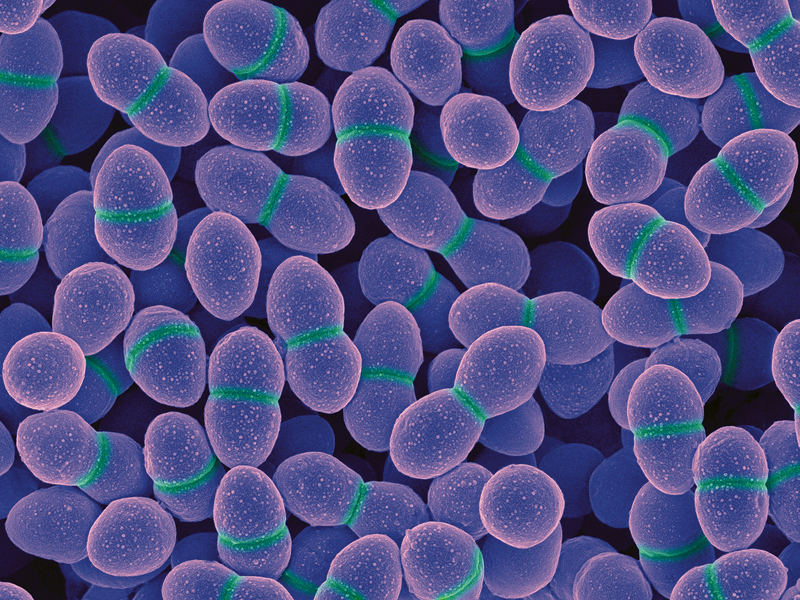
E. faecalis during cell division © Dennis Kunkel Microscopy, Inc.

These promising results suggest that trends in antibiotic resistance may be quickly reversed in some strains by switching to organic techniques. The researchers speculate that resistant bacteria persist on organic farms because, although organically raised broilers can be given no antibiotics from day 1 of life, the breeder farms where the eggs originate are under no restrictions on antibiotic use and may give mother hens antibiotic-laced feed. Hatcheries that subsequently supply day-old chicks to broiler farms also can inject eggs with antibiotics.

